# Organic Anode Materials for Lithium-Ion Batteries: Recent Progress and Challenges

**DOI:** 10.3390/ma16010177

**Published:** 2022-12-25

**Authors:** Alexander A. Pavlovskii, Konstantin Pushnitsa, Alexandra Kosenko, Pavel Novikov, Anatoliy A. Popovich

**Affiliations:** Institute of Machinery, Materials and Transport, Peter the Great Saint Petersburg Polytechnic University, Politechnicheskaya ul. 29, 195251 Saint Petersburg, Russia

**Keywords:** lithium-ion batteries, redox-active organic anode, organic anode materials

## Abstract

In the search for novel anode materials for lithium-ion batteries (LIBs), organic electrode materials have recently attracted substantial attention and seem to be the next preferred candidates for use as high-performance anode materials in rechargeable LIBs due to their low cost, high theoretical capacity, structural diversity, environmental friendliness, and facile synthesis. Up to now, the electrochemical properties of numerous organic compounds with different functional groups (carbonyl, azo, sulfur, imine, etc.) have been thoroughly explored as anode materials for LIBs, dividing organic anode materials into four main classes: organic carbonyl compounds, covalent organic frameworks (COFs), metal-organic frameworks (MOFs), and organic compounds with nitrogen-containing groups. In this review, an overview of the recent progress in organic anodes is provided. The electrochemical performances of different organic anode materials are compared, revealing the advantages and disadvantages of each class of organic materials in both research and commercial applications. Afterward, the practical applications of some organic anode materials in full cells of LIBs are provided. Finally, some techniques to address significant issues, such as poor electronic conductivity, low discharge voltage, and undesired dissolution of active organic anode material into typical organic electrolytes, are discussed. This paper will guide the study of more efficient organic compounds that can be employed as high-performance anode materials in LIBs.

## 1. Introduction

Rechargeable lithium-ion batteries (LIBs), one of the most successful commercialized secondary batteries, play a pivotal role in every aspect of our lives thanks to their suitable energy density, long service life, excellent rechargeability, and prolonged storage capacity. Nowadays, LIBs are an integral part of different consumer electronic devices, such as smartphones, laptops, watches, power banks, digital cameras, tablets, etc., and are expected to fuel plug-in hybrid electric vehicles and to be applied in smart grids in the near future. With worldwide industrial development and techno-economic growth, the energy density requirements for the above-mentioned energy-storage devices have continuously increased in the past few decades. In order to satisfy the ever-growing demand for consumer electronics and electric vehicles, achieving higher energy/power densities and longer cycle lives of LIBs has risen as a major issue for the development next generations of high-energy rechargeable Li-ion batteries.

In LIBs, the choice of electrode active materials is the dominant factor influencing the electrochemical performance of the batteries to a large extent. However, presently available commercial LIBs assembled with inorganic layered transition metal oxides, such as LiCoO_2_ (LCO), LiMn_2_O_4_ (LMO), LiFePO_4_ (LFP), LiNi_x_Co_y_Mn _1−x−y_ (NCM), etc. [1], as a cathode material and graphite or Si/C as the most commonly used anode material reach their limit in terms of sustainability, capacity, and output voltage. Graphite usually applied as a commercial anode in the current state-of-the-art LIB has poor rate capability and a low theoretical specific capacity of 372 mAh/g which significantly hampers the further development of LIBs [2,3,4]. Great research efforts have been put forth to find suitable alternatives to conventional graphite anodes with enhanced energy and power density. Thus, a variety of alloy-based materials (e.g., Sn, P, Ge, Si, Bi, Sb) and transition metal oxides (M_x_O_y_, M = Ni, Mn, Fe, Cr, Mo, Co, Nb, etc.) have been extensively investigated as high specific capacity LIBs anodes [5,6,7,8,9,10,11]. However, these anode materials for LIBs have poor electrochemical stability and fast capacity fading which impede their commercial application for Li-ion batteries.

Organic compounds with electroactive functional groups or moieties, either a polymer or small organic molecule, can undergo an electron transfer through a reversible electrochemical redox reaction. Therefore, they are suitable for the conversion and storage of chemical and electrical energy.

Compared with conventional inorganic anode materials, redox-active organic anode materials are endowed with obvious advantages, such as low cost, structural diversity, environmental friendliness, outstanding flexibility in their molecular design, low weight, and higher theoretical capacity, which can meet the rising demand for the energy density of next-generation battery systems [12,13,14,15]. In contrast to inorganic compounds, organic materials are mainly composed of such chemical elements that are widely distributed in nature (e.g., C, H, O, N, S). Thus, organic anode materials can be easily synthesized from renewable resources in facile steps [16] that have a minimal environmental footprint [17]. Moreover, both the tunable molecular structure and high structural diversity of organic compounds, unlike those of their metal-ion inorganic counterparts, allow for optimizing the electrochemical performance of organic electrode materials, such as redox activity and operating voltage in various metal-ion batteries [18,19,20,21].

For instance, the electronegativity of substituents in organic compounds could tune the redox potential. It was corroborated that the introduction of an electron-withdrawing group into the redox active organic molecule enhances the redox potential because the electron cloud around the redox center of the organic material is attracted toward higher electronegative groups, such as heteroatoms [22]. On the contrary, attaching an electron-donating group to the redox-active organic molecule reduces the redox potential [23]. It should be emphasized that some organic compounds enable the batteries to be exploited in extreme conditions, such as a high pH value, an extended temperature range from −70 to 150 °C, and the presence of oxygen [24]. With these advantages of organic electrode materials, LIBs based on organic electrodes have attracted considerable research interest and have made magnificent progress [20,24,25,26,27,28,29].

Due to the versatile properties of organic electrodes, various types of organic materials have been used in all kinds of rechargeable batteries, comprising all-solid-state batteries, nonaqueous sodium-ion, lithium-ion, potassium-ion, dual-ion batteries, multivalent-metal batteries, redox flow batteries, and aqueous batteries [24]. Furthermore, apart from small organic molecules, different polymers, polymer frameworks, and 2D organic materials can also be adopted as organic electrodes in rechargeable batteries [30,31,32,33].

For a long time, organic anode materials have received much less attention compared to their inorganic counterparts mostly because of their relatively low electronic conductivity and the tremendous success of inorganic anode materials in either application or research. Although there are already numerous reviews of organic electrode materials used in different kinds of rechargeable batteries [14,16,17,24,25,34,35,36,37], a review article that briefly, simply, and systematically introduces the fundamental knowledge of organic anode materials for LIBs is still absent.

Inspired by the advantages of organic electrode materials, we decided to write this review paper, as a brief introduction to the representative organic anode materials in LIBs, as well as the fundamental principles, state-of-the-art developments in organic anodes, and outlooks of these perspective materials for LIBs, aiming to cause more interest and innovation in the battery industry.

## 2. Fundamentals of Organic Anode Materials

### 2.1. Basic Components of an Electrochemical Cell

**Figure 1:**
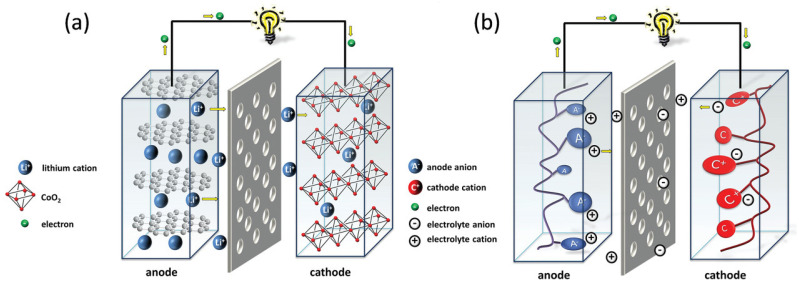
Schematic showing of the processes occurring during discharging of (**a**) traditional inorganic and (**b**) organic electrode-based Li-ion batteries. Instead of intercalation-based reactions in inorganic compounds, organic compounds undergo surface-based redox reactions. Reprinted with permission from Ref. [38], Copyright 2016, American Chemical Society.

Rechargeable (also called “secondary”) batteries consist of two electrodes, a cathode (or positive electrode) with high redox potential and an anode (or negative electrode) with lower redox potential, in contact with an electrolyte (either solid or liquid). The electrolyte system with low viscosity, high stability, and ionic conductivity at a large potential window is preferred. Furthermore, electrolytes must be inert towards both the cathode and active anode materials. The cathode and anode of the cell are connected through an external circuit. To avoid short circuits, the cathode is mechanically separated from the anode by an electronically insulating porous membrane (usually named separator). These are the widespread cell components for a battery. Figure 1 depicts a schematic of a typical battery design.

### 2.2. Mechanism of Charge Storage in Lithium-Ion Batteries and Working Principles of Organic Anode Materials

During the charging process of a battery, electrons are forced (by an applied potential or current) from the positive electrode to the negative electrode, oxidizing the cathode and reducing the anode. During the discharge process of a battery, electrons spontaneously flow in the opposite direction (from the negative electrode to the positive electrode), reversing the previous redox reactions. Thus, both positive and negative electrodes, with various redox potentials, must be capable of reversible electrochemical redox reactions. The difference in chemical potential between these reactions constitutes the operating voltage of the cell, also known as the open circuit voltage.

In contrast to inorganic electrode materials, with redox reactions that are based on the valence change of the elemental substance or transition metal, the redox reaction of organic electrode materials is based on the change in the charge state of the redox active organic species or an electroactive organic group. When an active electrode material changes its state of charge, oppositely charged ions in the electrolyte diffuse into the active material to charge compensate. Depending upon the potential of this redox process, organic materials can either be suited for use as a cathode or anode active material. The polarized state of organic active material is used for interaction with the mobile ion.

In other words, a reversible metal ion intercalation-based electrochemical redox reaction is employed to store the charge in conventional inorganic-based electrode materials. In organic electrode materials, a change in the charge state of the redox active moieties is utilized to store the charge. During the charge and discharge electrochemical process, the inorganic-based electrode structure is stabilized by the change in the oxidation states of the transition metals from inorganic cathode materials for LIBs, while in organic electrode material, the polarized state of organic active material is commonly balanced by its interaction with metal ions from the electrolyte.

### 2.3. Types of Organic Anode Materials and Their Advantages

**Figure 2:**
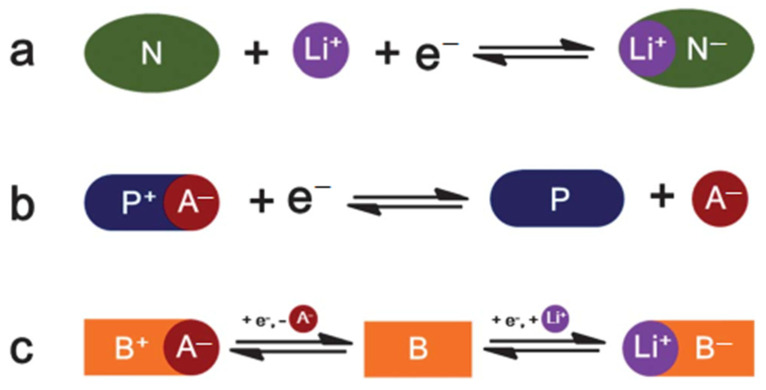
**The** reversible redox reaction of three types of redox-active organic compounds: (**a**) N-type; (**b**) P-type; (**c**) bipolar. The anion of the electrolyte is denoted as A^−^. Used with permission from the Royal Society of Chemistry, from Ref. [39]; permission conveyed through Copyright Clearance Center, Inc.

According to different redox reactions of organic electrode materials, they can be generally classified as N-type, P-type, or bipolar (Figure 2). N-type organics obtain a negative charge (N^−^) from the original neutral state (N) during the electrochemical reduction reaction and revert to their initial state by oxidation. P-type organic compounds (P) are oxidized during the redox process yielding positively charged cations. B-type (or bipolar) organics are another type of organic compound, for which the initial neutral state (B) can be either oxidized to a positively charged state (B^+^) or reduced to a negatively charged state (B^−^), which depends on the applied voltage. B-type organic compounds can be also considered N- or P-type organics since, in general, only half of the reaction takes place on one electrode.

In the electrochemical oxidation reaction of P-type or reduction reaction of N-type, anion (A^−^) or cation (Li^+^) are, respectively, required to neutralize the positive charge of P^+^ or the negative charge of N^−^. In the reverse redox reaction, lithium cations or A^−^ anions will move back from the electrode to the electrolyte.

The noticeable difference between N-type organic materials and inorganic intercalation compounds is that lithium ions can be substituted by other alkali metals, such as K^+^ and Na^+^ or even H^+^ cations, which will not significantly influence the electrochemical behavior of the material. Inorganic intercalation compounds, on the contrary, are very sensitive to the ionic radius of the cation.

For P-type organic materials, many kinds of anions can be adopted as A^−^, e.g., NO_3_^−^ and Cl^−^ in aqueous electrolytes, or TFSI^−^, PF_6_^−^, BF_4_^−^, and ClO_4_^−^ in non-aqueous electrolyte.

B-type organic compounds can act simultaneously as an anode and cathode active material. For instance, Yang and coworkers [40] reported such a bipolar organic compound as polyparaphenylene, which has a large potential gap of 3.0 V and electrochemical reversibility for the electrochemical p- and N-type redox reactions. They indicated that this bipolar redox-active polymer is the most attractive candidate for application in all-organic batteries with the same cathode and anode active material. The findings demonstrated that polyparaphenylene could be either p-doped with a specific capacity of 80 mAh/g at high potentials of >3.9 V or n-doped with a superior specific capacity of 350 mAh/g at a quite low potential region of <1.5 V [40].

### 2.4. Advantages of Organic Anode Materials

**Figure 3:**
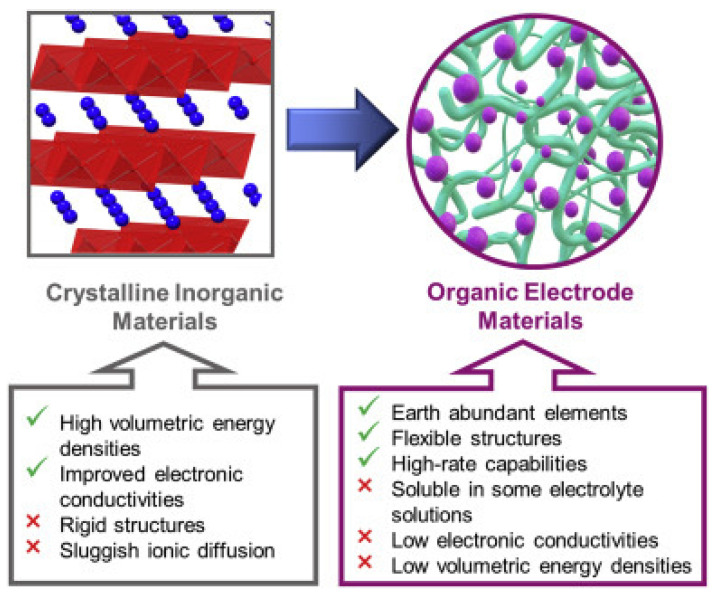
Schematic illustration of the unique properties of organic electrode materials which make them particularly attractive for use as high-performance electrode materials in rechargeable LIBs [25]. Ref. [25] is an open-access article distributed under the terms of the Creative Commons CC BY license, which permits unrestricted use, distribution, and reproduction in any medium provided the original work is properly cited.

It should be noted that in traditional inorganic intercalation-based electrode materials, lithium-ion insertion leads to the transformation of the cathode lattice structure. This result in heat generation and slow reaction kinetics during the charge/discharge process. At the same time, fast insertion and de-insertion of lithium-ion may result in the structural degradation of the cathode material. These challenges substantially affect both the rate and cyclic performance of the typical LIBs, impeding their role in long-life and high-power applications. In contrast, organic electrode materials undergo fast and simple redox reactions without structural degradation of the electrode active material. Thus, they exhibit excellent cyclic and rate performance. Moreover, as previously stated, inorganic intercalation-based materials are very sensitive to the ionic radius of cation, whereas, in organic electrode materials, lithium cation can be easily substituted with various alkali metal cations with a bigger ion radius, such as Na^+^, K^+^, etc., without any significant fade of the capacity of batteries. In addition, both the larger interlayer spacings and greater flexible structures of organic electrode materials (Figure 3) afford faster ion diffusion and reduced structural and volumetric changes upon charge/discharge processes, enhancing rate capabilities [25].

## 3. Organic Anode Materials Based on Organic Carbonyl Compounds

To date, different kinds of redox-active moieties, such as phenazine, carbonyl, triazine, imide, and aromatic ring structures, have been acknowledged as the main functional groups in redox-active organic anodes [41,42,43,44,45,46,47,48]. Additionally, conducting polymers, thioethers, organodisulfides, conjugated carbonyl compounds, radical polymers, and other organic compounds have been reported as anode active materials [49,50,51,52,53,54,55,56,57,58,59].

Among all reported redox-active organic compounds, carbonyl compounds, such as imides, quinones, carboxylates, ketones, anhydrides, and their derivatives have become a class of attractive organic materials for use as potentially high-capacity anode or cathode materials for LIBs during the past decades [58,60,61,62,63,64,65]. This is because of their highly active redox carbonyl centers providing wide structural diversity, higher reversible capacity, and fast kinetics. For instance, the simplest quinone (1,4-benzoquinone) can provide a reversible capacity of nearly 500 mAh/g [66]. Carbonyl functional groups (C=O) can be found in numerous organic compounds with different forms, while most of the investigated carbonyl-based organic electrodes are primarily based on either the derivatives of aromatic carboxylic acid or quinone [67,68,69].

According to the literature, conjugated carboxylates, aromatic imides, quinones, and aromatic anhydrides are the four categories of conventional organic carbonyl electrode materials which have been successfully applied in LIBs [47]. Nearly all carbonyl compounds are N-type electrode materials and are reduced during the charge/discharge process, forming a negatively charged anion. Their redox mechanism can be presented as a nucleophilic addition reaction of electrochemically active C=O functionalities, in which the enolization reaction is the main step [39].

Although most quinone derivatives have been primarily utilized as cathode materials for LIBs (because these quinone systems have voltages of ≳2.0 V versus Li/Li^+^, typical for LIB cathodes), some conjugated carbonyl materials with low redox potentials could be used as negative electrodes for LIBs, which stems from the significant charge repulsion interaction between CO− moieties [70]. Carboxylates with electron-donating groups (such as -OLi) also reveal a redox reaction at a very low potential and, thus, they can be also utilized as anode materials [71].

Lithium *trans-trans-*muconate (Li_2_C_6_H_4_O_4_) and lithium terephthalate (Li_2_C_8_H_4_O_4_) were the first two examples of carbonyl anode materials for LIBs proposed by Tarascon’s research group in 2009 [71]. Remarkably, both Li_2_C_6_H_4_O_4_ and Li_2_C_8_H_4_O_4_ as a negative-electrode material exhibit very flat discharge plateaus at 1.4 and 0.8 V versus Li/Li^+^, respectively, which allows the use of a cheaper aluminum current collector for the anodes of LIBs. A noteworthy advantage of the organic anode material based on these two conjugated dicarboxylates is their higher thermal stability over typical carbon negative electrodes in typical carbonate electrolytes (1 M LiPF_6_ in ethylene carbonate (EC)/dimethyl carbonate (DMC)), which should lead to safer Li-ion batteries [71].

Li_2_C_6_H_4_O_4_ revealed a reversible capacity of 170 mAh/g with 73.5% (≈125 mAh/g) capacity retention after 80 cycles at 0.1C rate charge/discharge, while Li_2_C_8_H_4_O_4_ delivered a greater reversible capacity of 300 mAh/g with 78% (≈234 mAh/g) capacity retention after 50 cycles at a current density of 15 mA/g [71].

**Figure 4:**
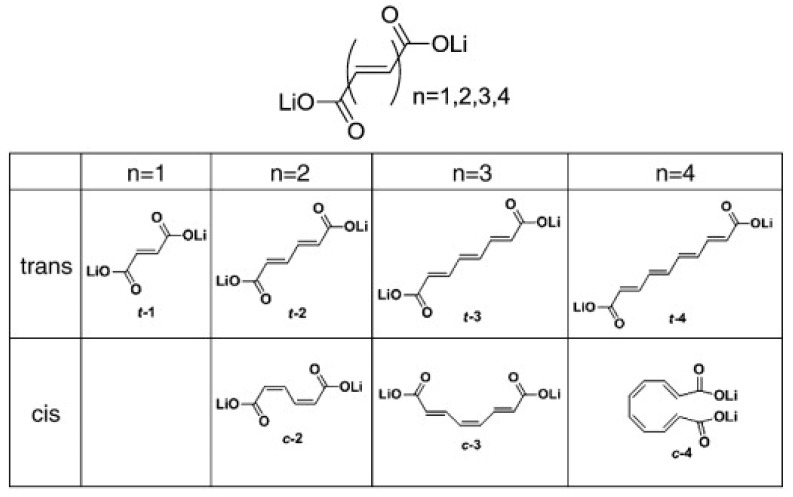
Chemical structures of the conjugated carboxylates with conjugation pathways of 2, 3, and 4 units. Reprinted from Ref. [72], Copyright 2010, with permission from Elsevier.

It should be emphasized that only the trans versions of conjugated carboxylates LiO_2_C(CH=CH)_n_CO_2_Li with n = 2, 3, and 4 (Figure 4) showed reversible electrochemical reactivity towards lithium [72].

Ethoxycarbonyl-based lithium salt (Li_2_C_18_H_12_O_8_) delivered a capacity of 125 mAh/g in the initial cycle and about 110 mAh/g in the 50th cycle over two main voltage plateaus at ~1.96 and 1.67 V [68].

**Figure 5:**
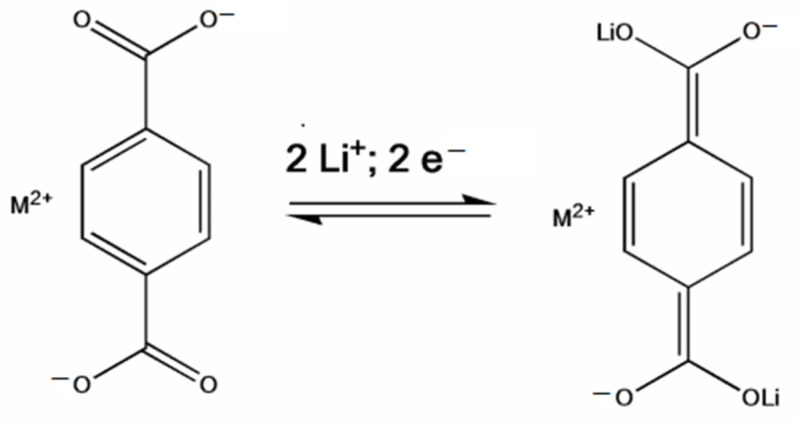
Schematic depicting the lithium storage/release mechanism of carbonyl-based terephthalates at the molecular structure level. Reprinted from Ref. [73], Copyright 2016, with permission from Elsevier.

Wang and co-workers [73] prepared alkaline earth metal terephthalates MC_8_H_4_O_4_ (M = Ca, Sr, Ba) via eco-friendly and facile displacement reactions and adopted these organic compounds as anodes for LIBs. The scheme of the lithium storage mechanism of carbonyl-based terephthalates on the level of molecular structure is presented in Figure 5.

In the discharge process, the calcium terephthalate displays a flat plateau at nearly 0.8 V vs. Li/Li^+^. Overall, metal terephthalates produced by Wang and colleagues exhibited a reversible capacity of nearly 130 mAh/g at 0.2 A/g. They also found that the main factors determining the various electrochemical properties (operational voltage, rate performance, cyclic lifetime, and specific capacity) of the obtained organic anodes are the electrostatic interactions between the terephthalate anions and metal cations leading to the formation of metal-organic frameworks (MOFs) with high crystalline structure [73].

Organic metal salts, such as lithium tannic acid (LiTA), have been also applied as advanced energy storage anode materials for LIBs. The LiTA anode shows a reversible capacity of 133.5 mAh/g at a current density of 100 mA/g and maintains 100.5 mAh/g after 100 charge–discharge cycles. Besides, LiTA demonstrates excellent rate properties, surprisingly long cycle performance, and high coulombic efficiency resulting from reversible and stable redox reactions between C-O, and C=O chemical bonds [74].

Xiao and co-workers [75] synthesized copper maleate hydrate (CMH) and utilized this organic material as an organic anode material for rechargeable LIBs for the first time. This small molecular organic compound demonstrated superior electrochemical performance. It exhibited an initial reversible capacity of 404.6 mAh/g at a current density of 0.2 A/g and could maintain a reversible capacity of nearly 383.2 mAh/g after 250 cycles [75].

Table 1 summarizes the above-stated information about typical organic anode materials for LIBs based on carbonyl compounds.

It should be noted that all carboxylate-based derivatives featuring straight chains could only achieve one-Li uptake, thus resulting in low capacity (less than 170 mA/g). In sharp contrast, Li_2_C_8_H_4_O_4_ consisting of a terephthalate system that does not have a straight carbon chain could realize a full two-Li uptake, giving a stable specific capacity of nearly 300 mAh/g [71]. Therefore, further efforts were primarily made in enhancing the electrochemical performance of the terephthalate systems, including carbon coating, nanosizing, constructing metal-organic frameworks, modification of the aromatic core, etc. [78,79,80,81].

## 4. Organic Anode Materials Based on Covalent Organic Frameworks (COFs)

Covalent organic frameworks (COFs) materials are a subclass of crystalline microporous organic polymers featuring atomically precise self-assembled two- (2D) or three-dimensional (3D) extended structures with ordered nanopores, strong covalent bonds between lightweight elements (C, N, O, B, etc.) of rigid building blocks, and periodic skeletons [82,83]. Apart from cathode materials, COFs have been heralded as perspective organic anode materials [35].

Compared with typical polymers and small organic molecules, COFs are more preferable candidates for use as electrode materials in LIBs owing to their structural diversity, porous structure, framework tunability, low electrode volume change, large specific surface area, and functional versatility [20,35,84,85,86].

The capacitance capacity of COFs is caused by their porous structure, whereas their specific capacity comes from the insertion of lithium ions in the active redox site. A large molecular weight of COFs coupled with the robustness of their framework inhibits the dissolution of this material in electrolytes, thus enabling a stable cycle performance. At the same time, the porous framework structure reduces the path of ion diffusion, leading to enhanced kinetics of lithium ions [35].

Among the diversity of organic framework materials, COFs based on nitrogen-containing active sites have gradually garnered the merited attention of researchers in recent years [87].

Yang and coworkers [88] reported the first example of an organic 2D COF material for an anode of LIB. Their work paved the way for the utilization of COFs in the next-generation high-capacity LIB. Researchers designed 2D COFs based on covalently linked porphyrin with 4-thiophenephenyl groups (TThPP) and used the TThPP films grown on the copper foil without adding any binders as the anode of LIBs. Due to the alignment of 2D polyporphyrin nanosheets, this material demonstrated high electronic conductivity, long cycle lives, insolubility, high specific capacities (up to 666 mAh/g), and outstanding rate performances. The high capacity and excellent rate performance of TThPP have been explained by the presence of many sites for efficient adsorption of lithium atoms and the existence of both open nanopores holding electrolyte and short-ended paths for the rapid lithium-ion diffusion in the TThPP structure [88].

Bai and coworkers [89] presented two fully conjugated porous COFs as the organic anode of LIB, which demonstrated stable long life (500 cycles) and excellent specific capacity of ca. 700 mAh/g. Such superior charge–discharge performance corroborated the high potential of COFs to be utilized for eco-friendly energy storage. More importantly, utilizing conjugated COFs not only avoids complicated synthesis of typically conjugated polymers and doped or multilayered hybrids but also embodies their outstanding properties, such as flexibility, high electrochemical activity, structural diversity, etc., into energy conversion applications.

**Figure 6:**
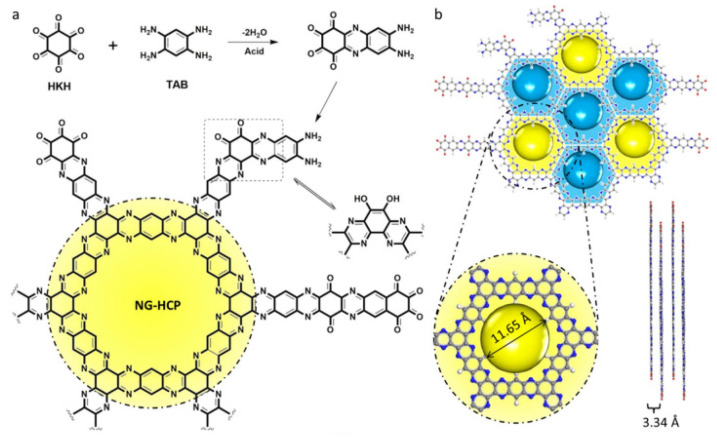
(**a**) Synthetic route of obtaining HG-HCP by a dehydration–condensation reaction between hexaketocyclohexane (HKH) octahydrate and 1,2,4,5-tetraaminobenzene (TAB) tetrahydrochloride in NMP solution with sulphuric acid as a catalyst and a schematic illustration of the alternative pristine NG-HCP structure, where many excessive amino and carbonyl functional groups exist at the edge; (**b**) molecular configuration of a single layer of the NG-HCP with a pore size of 11.65 Å and a packing distance of 3.34 Å. Reprinted from Ref. [90], Copyright 2017, with permission from Elsevier.

Zhang’s research group [90] opened a window for the promising applications of high-performance covalent organic framework (COF) structured polymers in flexible electronics and optoelectronic devices in the future anode market. They produced a two-dimensional (2D) nitrogen-rich graphene-like holey conjugated polymer (NG-HCP) through nanoengineering (Figure 6) and evaluated the as-prepared NG-HCP nanosheets as the anode material of Li-ion batteries. The findings demonstrated that the obtained NG-HCP nanosheets displayed an extremely high initial charge capacity of nearly 1320 mAh/g at a current density of 20 mA/g in the voltage range of 3–0 V vs. Li^+^/Li, without noticeable capacity loss during the first 20 cycles, and ultralong-term cycling life of 600 cycles [90]. Remarkably, after 230 cycles, the specific capacity of the NG-HCP after nano-engineering remained at a high value of nearly 1015 mAh/g under a current density of 100 mA/g. For comparison, the as-fabricated NG-HCP for the LIB anode without nano-engineering exhibits a specific capacity of less than 300 mAh/g. On the one hand, this outstanding rate performance of NG-HCP after nanoengineering is achieved due to both the large specific surface area and increased reaction contact area, which promote the charge-transfer reaction. On the other hand, the doping of hetero-atoms (such as nitrogen atoms) into the chain of the conjugated COF structure provides the increased electrochemical reactivity of the NG-HCP material, resulting in the superior performance of the electrode material. To sum up, the good dispersity of NG-HCP nanosheets with high electrical conductivity and the synergistic effects of N-doping and high porosity make NG-HCP nanosheets suitable as excellent anodes for LIBs.

**Figure 7:**
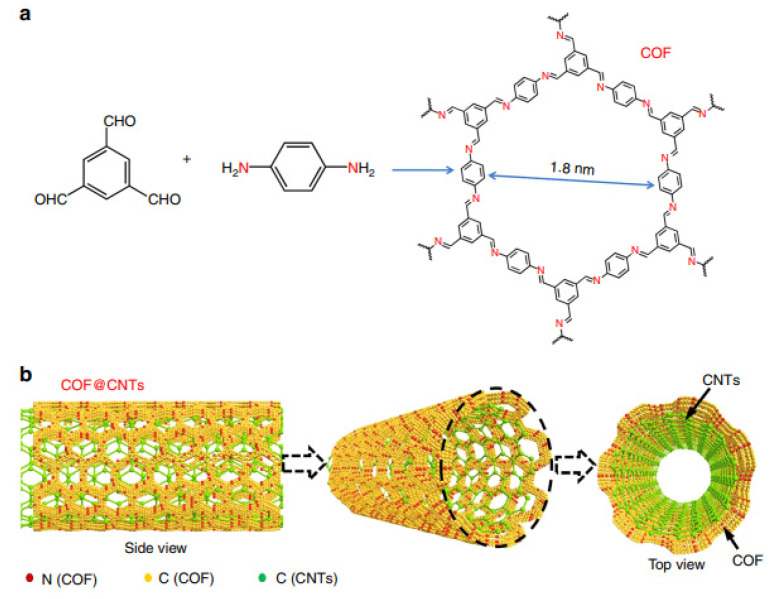
Preparation of the COF and COF@CNTs composite. (**a**) Preparation of COF with a 2D structure and an average pore size of 1.8 nm; (**b**) graphical representation of the COF@CNTs anode material with few COF layers covered on the exterior surface of carbon nanotubes [91]. Ref. [91] is an open-access article distributed under the terms of the Creative Commons CC BY license, which permits unrestricted use, distribution, and reproduction in any medium provided the original work is properly cited.

Lei and coworkers [91] synthesized a composite electrode material (COF@CNTs) through the controllable growth of few-layered imine-based 2D COFs on the exterior surface of carbon nanotubes (CNT) for application as the anode of LIBs. Imine-based C=N functional group coupled to COF is shown in Figure 7a, while the graphical illustration of obtaining COF@CNTs composite featuring few COF layers covered on the exterior surface of CNTs is illuminated in Figure 7b.

Remarkably, upon activation, COF in the composite (COF@CNTs) proposed by Lei et al. [91] as an organic anode material for LIBs delivers an extremely large reversible capacity of 1536 mAh/g (corresponding to the 14-lithium-storage mechanism for a COF monomer), which even exceeds the theoretical capacity (700–1000 mAh/g) of conventional Sn-based anodes and such inorganic anode materials as transition metal oxides (Co_3_O_4_, Fe_2_O_3_, CuO, NiO, etc.). More importantly, after 500 cycles, the reversible capacity of COF@CNTs material is sustained at a high value of 1536 mAh/g under a current rate of 100 mA/g.

**Figure 8:**
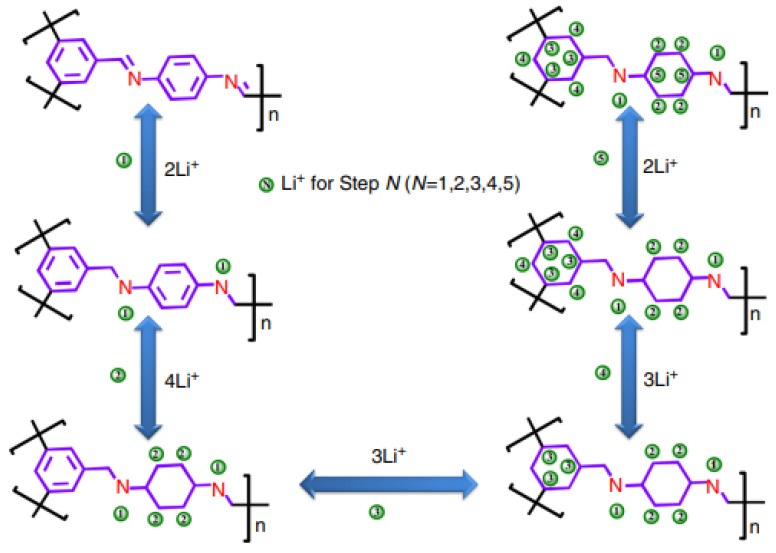
Schematical representation demonstrating the stepwise lithium-storage mechanism for a COF monomer in the COF@CNTs composite anode material [91]. Ref. [91] is an open-access article distributed under the terms of the Creative Commons CC BY license, which permits unrestricted use, distribution, and reproduction in any medium provided the original work is properly cited.

The underlying reason for the ultrahigh capacity of COF@CNTs composite negative electrode might be explained by the efficient utilization of active centers of the COF@CNTs anode for lithium-ion storage, which includes one lithium ion per C=N functional group and six lithium ions per benzene rings. Unlike the conventional C=O or C=N chemical bonds which have limited lithium-ion storage capability, the six lithium ions per benzene ring provide a much higher specific capacity during the lower voltage window [91]. This 14-lithium storage mechanism based on the five-step lithium-ion insertion/extraction process for a COF monomer in the COF@CNTs anode is presented in Figure 8.

Lei and coworkers [92] produced two types of triazine-based covalent organic nanosheets (CONs) with various pore sizes: Schiff base networks-1 (SNW-1) with a pore size of 0.5 nm and covalent imine network-1 (CIN-1) with a pore size of 2 nm. In order to obtain increased Li-ion storage capacity, the as-exfoliated CONs were further composited with carbon nanotubes (CNTs) (denoted as SNW-1/CNT and CIN-1/CNT composites) to expose more active sites for lithium storage and enhance the ions/electrons diffusion. It is worth noting that the obtained SNW-1/CNT and CIN-1/CNT composites are porous polymers connected by covalent chemical bonds, which can be also considered amorphous COF materials having disordered pore structures. After modification of these composites by mechanical stripping, exfoliated E-SNW-1/CNT and E-CIN-1/CNT exhibit outperforming lithium storage charge–discharge performances thanks to the facilitated surface-control kinetic coupled with the thin-layered 2D structures of these exfoliated CON materials. When employed as an organic anode material for LIBs, E-SNW-1 and E-CIN-1 materials demonstrate extremely high reversible capacities after conducting 250 cycles: 920 mAh/g and 1005 mAh/g, respectively (at a current density of 100 mA/g). Moreover, the mechanism of Li-storage has been found to have an intriguing 11 (for E-CIN-1/CNT electrode) and 16 (for E-SNW-1/CNT electrode) electrons involved in the electrochemical redox reactions, associated with not only typical organic functional groups (-NH- or C=N groups) but also unusual functional groups, such as triazine, piperazine, and benzene rings.

In 2021, Wu and coworkers [93] reported a high-rate anode material for LIBs based on redox-active piperazine-terephthalaldehyde COF (denoted as PA-TA) featuring ultra-large interlayer distance. Their research indicates that increasing the interlayer distance of COFs by a reasonable molecular design strategy would be of particular importance to producing high-performance anode materials for LIBs. The proposed PA-TA COF anode material by Wu and coworkers [93] exhibited remarkable cycling and rate performance delivering a high lithium storage capacity of 543 mAh/g even after 400 charging-discharging cycles at a current density of 1 A/g. Most importantly, the PA-TA COF anode could display a reversible capacity of 207 mAh/g even at a high current rate of 5 A/g.

It is worth noting that there are only several COF-related compounds that have been proposed as potential electrode materials for Li-ion batteries due to their redox-active centers with lithium ions, such as C=O and C=N functional groups, and micro/mesoporous structure [88,89,94,95,96]. The COF materials bearing carbonyl moieties are commonly investigated as cathodic materials for LIBs based on the lithium redox reaction with C=O species [94,95,96]. The existence of aromatic heterocyclic functional groups and/or imine moieties promotes the application of COF materials as the anode materials for LIBs [88,89].

The key electrochemical properties of COF-based organic anode materials for LIBs listed above are compared and summarized in Table 2.

## 5. Metal-Organic Framework-Based Anode Materials for LIBs and Their Practical Applications in Lithium-Ion Full Cells with Different Cathode Materials

Among the novel electrode materials, metal-organic framework (MOF) nanostructures have aroused intensive research interest as anodes for LIBs due to their extremely high surface area, controllable structures, large porosity, and presence of metal ions with redox activities which might be profitable to large lithium-ion accommodation space as well as an effective pathway for lithium ion and electron transport. Typically, MOFs consist of two major components: organic linkers (ligands) containing functional groups to build an open framework, and the metal ions (metal clusters) as redox-active centers which coordinate with organic ligands forming one- (1D), two- (2D), or three (3D)-dimensional structures. In particular, transition metals with high redox activity are used as metal ions of MOFs.

To date, numerous MOF-based compounds have been reported for various components of LIBs including cathodes, separators, electrolytes, and anodes. Many efforts have been made to construct MOF-based anode materials for LIBs in order to achieve higher capacity when working in LIB full cells with commercial cathode materials. Thus, in this section, different lithium battery systems composed of MOF-based anode materials are discussed.

In 2006, Chen and coworkers [97] first proposed a Zn-based MOF, Zn_4_O(1,3,5-benzenetribenzoate)_2_, named MOF-177, as an anode material for LIBs. They confirmed that different morphologies of this MOF can be regulated by a solvothermal route. However, the electrochemical performance of as-synthesized MOF-177 is found not satisfactory for practical application in reversible LIBs since the cycling capacity of MOF-177 was limited [97]. Besides that, in terms of anodes in LIBs, other valence metals have been used to design a variety of MOFs, such as Co-based MOF-71@300N [98], Cu-based MOF-199 [99], carbon-coated ZnO quantum dots-based MOF—MOF-5 [100], organic-coated ZIF-8 nanocomposites [101], Fe-based MIL-88 [102], Co-based zeolitic imidazolate frameworks—ZIF-67 [103], etc. However, all of these listed MOF-based anode materials still suffer from either poor stability or limited capacity in the first several charge–discharge cycles.

Song and coworkers [104] fabricated for the first time one-dimension (1D) cobalt-based MOF nanowires (named CoCOP) with a facile hydrothermal reaction and explored the as-prepared CoCOP nanowires as the anode material for coin-type full LIBs with commercial LFP cathode powders. They found that typical coin-type full Li-ion batteries with the CoCOP nanowires anode material and commercial LFP cathode material demonstrated a high capacity of 138 mAh/g at a current rate of 0.1C. Moreover, these LFP|| CoCOP full Li-ion batteries exhibited rapid charge–discharge capability and outstanding cyclability with a good capacity retention of 83% after conducting 300 cycles at a 1C charge–discharge rate, between 0.4 and 3.8 V [104].

In follow-up work, Song and coworkers [105] investigated a series of metal inorganic-organic hybrid composites (M-IOHCs, where M is Mn, Co, or Ni) as anode materials for Li-ion storage. Among all tested M-IOHC anode materials, Ni-IOHCs exhibited higher lithium storage capacity and showed an excellent power density of 297 W/kg at an energy density of 32 Wh/kg when assembled with a commercial LiFePO_4_ cathode powder in a full coin-type cell system.

To achieve lower toxicity and lower cost, some researchers adopted Fe-based MOFs as anode materials for LIB full cells. For instance, Shen and coworkers [106] applied [Fe_3_O(BDC)_3_(H_2_O)_2_(NO_3_)]_n_ (named as Fe-MIL-88B) as a Fe-based MOF anode material for CR2032 full cells with commercial LFP powders as a cathode material. The full cells demonstrated an initial discharge capacity of 108 mAh/g at a current rate of 0.2C in a voltage cutoff of 0.4–3.8 V, and a large capacity of 89.8 mAh/g was retained after 100 repetitive cycles at a 0.25C charge–discharge rate. Overall, the findings obtained by Shen and coworkers [106] revealed that this Fe-MOF material is a prospective candidate for stable Li-ion insertion/de-insertion electrochemical processes.

Similarly, Sharma and coworkers [107] used another Fe-MOF configured with two ligands consisting of naphthalene dicarboxylic acid (H_2_NDC) and terephthalic acid (H_2_BDC) as an anode material for a full (coin) cell device assembled with LCO cathode material coated on aluminum foil. Impressively, this LCO||Fe-MOF LIB full cell displayed a significant energy density of 360 Wh/kg (which is equivalent to a reversible capacity of 120 mAh/g) at a current density of 500 mA/g, with high cycling stability up to 1000 cycles.

It should be noted that the above-mentioned MOF anode material achieved interaction with lithium-ion through the valence change of its constituent transition metal. However, some conductive MOFs can interact with lithium-ion without the participation of their constituent transition metals in the lithiation/de-lithiation cycling processes.

For instance, Guo and coworkers [108] explored for the first time a bottom-up solvothermal technique to produce a one-dimensional (1D) Cu-based conductive MOF with 2,3,6,7,10,11-hexahydroxytriphenylene as the organic ligand (denoted as Cu-CAT) and found it as a competitive high-rate anode material for robust LIBs. A Cu-CAT nanowires-based full cell constructed with NCM811 cathode powder demonstrated outstanding cycling behavior, as well as a high energy density of ca. 275 Wh/kg. Remarkably, many nanowires of the Cu-CAT material of several micrometers in length are randomly connected together to build a three-dimension (3D) cross-linked porous network, which promotes the diffusion of electrolyte ions and convenient penetration of Li-ions.

In 2020, Weng and coworkers [109] reported for the first time such a Zn-based conductive MOF, [Zn_2_(py-TTF-py)_2_(BDC)_2_]·_2_DMF·H_2_O, with terephthalic acid (H_2_BDC) and 2,6-bis(4′-pyridyl)tetrathiafulvalene (py-TTF-py) as organic ligands and tested this material as an anode material for LIBs. The full cell of this Zn-based MOF with NCM622 cathode powder displayed a large reversible capacity of 131.9 mAh/g at a current rate of 100 mA/g with high Coulombic efficiency of 99.45% after 70 repetitive discharging/charging cycles. Moreover, this NCM622||[Zn_2_(py-TTF-py)_2_(BDC)_2_]·2DMF·H_2_O LIBs full cell demonstrated tolerance to high-current operation. Remarkably, the valence of Zn is not changed during the discharge/charge process, i.e., Zn^2+^ ions are not involved in the reversible electrochemical reaction occurring in LIBs, while Li-ions bond with sulfur atoms of the tetrathiafulvalene (denoted as TTF) moiety which provide the inserting sites of Li-ions during the discharging process.

It is worth noting that MOF-based composites prepared by an in situ growth strategy can also be used as organic anodes for LIBs. For instance, Wei and coworkers [110] used reduced graphene oxide (rGO) as a hard template for in situ growth of a fluorine-doped Co-based MOF with molecular formula Co_2_[F_x_(OH)_1−x_]_2_(C_8_O_4_H_4_), named as F-Co-MOF, using a facile solvothermal reaction. Owing to the synergistic effect of high conductive rGO networks and the F-Co-MOF structure, an outstanding reversible capacity of 162.5 mAh/g at a current rate of 200 mA/g after 300 repetitive cycles was achieved when this F-Co-MOF/rGO composite was adopted as an anode active material for a Li-ion full cell device assembled with LFP cathode material.

Based on the ultrahigh theoretical specific capacity of silicon (Si) and the large porosity of MOFs, some studies have focused on combining MOFs with Si through an in situ self-assembly technique to obtain higher cycling stability, relieve vigorous volume expansion of a silicon anode during the Li^+^ insertion/extraction process, and achieve higher lithium storage capacity.

For instance, Zhou and coworkers [111] encapsulated Si nanoparticles with well-designed rod-like cross-linking Sn-based MOFs containing *o*-phthalic acid as organic linkers through an in situ self-assembly strategy. Thanks to the distinctive hybrid structure with remarkable synergistic effects based on Sn-based MOFs and Si nanoparticles, the Si@Sn-MOF composite delivered a discharge capacity of ca. 117.7 mAh/g after discharging/charging 150 cycles when tested as anode material for a Li-ion full (coin) cell device assembled with NCM622 cathode material at a fixed potential window from 2.8 to 4.3 V [111].

Likewise, Nazir and coworkers [112] reported a facile method for preparing a Si anode material coated with a conductive two-dimension (2D) Cu-based MOF named Cu_3_(HITP)_2_, in which HITP = 2,3,6,7,10,11-hexaiminotriphenylene, through an in situ growth. They evaluated this anode material in a Li-ion full cell with LCO cathode powder. Thanks to the improved ionic and electronic conductivity, as well as prospective volume expansion buffer, the full cell composed of LCO cathode powder and this Cu-MOF-coated Si anode material supplied high reversible capacities at various current densities: 1267 mAh/g at a 0.5C rate and 1105 mAh/g at a 1C charge–discharge rate.

It is worth noting that, unlike pristine MOFs, MOF derivatives possess better practical application potential in LIBs owing to their higher cycling stability and conductivity. Some studies have shown that the metals or other constituent heteroatoms of MOFs can be evenly distributed in the porous organic (carbon) matrix through the pyrolysis of the organic framework.

For instance, Yu and coworkers [113] synthesized MOF derivatives such as NiSb⊂CHSs, in which NiSb−embedded carbon hollow spheres. Firstly, they obtained the black Ni⊂CHSs precursor via the thermal treatment of a Ni-based MOF under an H_2_/Ar atmosphere. Then, the as-prepared Ni⊂CHSs precursor was dispersed in the ethanol solution of SbCl_3_ salt by virtue of ultrasonication. Finally, the black NiSb⊂CHSs product was collected by centrifugation, washed, and dried under a vacuum. The NiSb⊂CHSs were used for the first time as organic anode materials for LIBs, demonstrating remarkable rate capability, perfect cycling performance, and high specific capacity. When tested at a high voltage operation of nearly 3.5 V, the NiSb⊂CHSs||LMO full cell device displayed a high coulombic efficiency of ca. 99% and remarkable rate capability (210 mAh/g at a current density of 2 A/g) [113].

In 2021, Wang and coworkers [114] fabricated a Ni-Co-Sb/C nanosphere anode material for LIBs through a two-step “template sacrifice method” of calcination treatment of the Ni-Co-MOF nanosphere precursors. Due to the alloying mechanism, the Ni-Co-Sb/C nanospheres derived from Ni-Co-MOF delivered a high reversible capacity of 354 mAh/g at a current density of 100 mA/g even after 100 repetitive cycles when assembled with LCO cathode powder.

It should be noted that the sacrificial template method can also be utilized to fabricate Si-based anode material in which the in situ growth carbon possessing a porous structure generates a higher specific surface area. For instance, mesoporous silicon hollow nanocubes (denoted as m-Si HCs) were successfully produced by using such classic examples of MOFs as ZIF-8 as the sacrificial template [115]. The findings demonstrated that a full Li-ion cell consisting of LCO cathode material and m-Si HC-graphite anode material displayed outstanding cycle retention of 72% after 100 repetitive cycles at a 0.2C current rate. This excellent electrochemical performance corroborates that the applied template method is a facile and effective route to fabricate high-performance anode materials for LIBs [115].

Other than utilizing the sacrificial template method for producing Si-based organic anode material, using MOFs as a sacrificial template allows metal oxides with various properties and structures to be obtained by changing the calcination temperature and gas atmosphere.

For instance, Sun and coworkers [116] fabricated Co_3_O_4_ via both a two-step and one-step calcination of Co-based MOF precursor, respectively. Thanks to the 3D porous starfish-like structure, the Co_3_O_4_@N-C nanocomposite fabricated via a two-step technique delivered stable discharge capacity and could effectively buffer volume expansion during the charging/discharging process. Moreover, the Co_3_O_4_@N-C||LiFePO_4_ full Li-ion battery showed stable capacity retention of 95.3% after 100 repetitive cycles [116].

It is worth noting that the calcination of Co-based MOFs that have heteroatoms or other additives allows cobalt oxide composites to be obtained that give full play to each component for achieving superior performance. For example, a Zn-doped hollow core–shell nano-sized Co_3_O_4_, named as Zn-Co-Oxide, derived from ZIF-67 via the template method exhibited a remarkable and stable specific capacity of 1600 mAh/g at a current density of 1 A/g for 700 repetitive cycles in the Li-ion full coin cell assembled with NCM532 cathode powder [117]. Fei and coworkers [118] produced a one-dimensional (1D) bunched Ni-MoO_2_@Co-CoO-NC composite. Their synthetic strategy involved the room temperature growth of a ZIF-67 polyhedron onto NiMoO_4_·xH_2_O nanowires followed by a carbonation treatment stage under Ar/H_2_ atmosphere. Thanks to the synergistic effects of the variable metal and the well-designed morphology of Ni-MoO_2_@Co-CoO-NC composite, a Ni-MoO_2_@Co-CoO-NC||LiFePO_4_ full Li-ion battery exhibited a high energy density of 329 Wh/kg and excellent cycling performance.

The electrochemical properties of rechargeable Li-ion full cells constructed with MOF-based anode materials and commercial cathode powders are summarized and compared in Table 3.

As outlined in this section, the discharge/charge processes occurring in MOF-based anode materials are governed by conversion or intercalation charge-storage mechanisms. In the future, during the design of MOF-based anode materials, special attention should be paid to the following aspects:

1. Abundant redox active sites afforded by organic moieties or metal ion centers for the reversible electrochemical redox reaction;
2. Construction of conductive frameworks by importing heteroatoms and carbonization;
3. Adjustable porous frameworks for easier Li ions and electron transmission;
4. Utilization of the synergistic effect between various active sites of MOFs for next-generation LIBs with higher specific capacity.

## 6. Organic Compounds with Nitrogen-Containing Groups as Anode Active Material for LIBs

In recent years, to continue to investigate more organic materials that can be employed for rechargeable electrodes, the element N, which possesses an electronegativity close to that of oxygen, has attracted enormous attention.

Organic compounds with nitrogen-containing groups (OCNs) are regarded as some of the most promising organic electrodes because of their outstanding electrochemical properties, versatility, structural diversity, and adjustability [87].

To date, almost all organic anode compounds contain either an alicyclic ring group or a harmful aromatic ring, which leads to high toxicity. Such organic anode materials are undoubtedly not suitable for developing environmentally friendly and toxin-free LIBs. Zhu and coworkers [54] proposed the first linear chain-type organic compounds without benzene groups, 2,2′-azobis(2-methylpropionitrile) (AIBN) and 2,2′-azobis(2-methylpropionamidine) dihydrochloride (AIBA), as novel commercially available organic anode materials for LIBs based on the azo group. Another benefit of these low-toxic AIBN and AIBA anode materials is that they are renewable, sustainable, and inexpensive. Interestingly, AIBN-anode material contains one azo group which acts as a reversible electrochemical active site for the reversible insertion and extraction of lithium ions, whereas AIBA-anode has two active sites of azo (N=N) and imine (C=N) groups, confirmed with in situ Raman and ex situ Fourier transform infrared spectroscopy. The specific capacity of the AIBN anode in LIB was approximately 100 mAh/g at a current density of 10 mA/g from the 2nd to the 100th cycle [54].

Ye and coworkers [119] synthesized a highly conjugated polymeric Schiff base (PSB) through a facile solid-phase reaction. The obtained PSB anode exhibits a high specific capacity of 175 mAh/g at a current density of 10 mA/g and outstanding cyclic performance. Most importantly, due to the suppressed dissolution of the as-prepared PSB into the organic electrolyte, great stable cycling with a capacity retention of 90% was achieved after 100 repetitive cycles. Such success opened up new avenues for developing more Schiff base polymers and producing more promising and higher-capacity organic anode materials for LIBs.

Man et al. [120] synthesized a highly conjugated organic framework, poly(imine-anthraquinone) (PIAQ), through an environmentally friendly and green in situ solvothermal condensation reaction of 2,6-diaminoanthraquinone (2,6-DAQ) and p-phthalaldehyde (PPD) and adopted this material as the anode material of LIBs. Based on DFT calculations coupled with experimental investigations, Man and coworkers [120] proposed a 16 Li-storage mechanism of PIAQ. Intriguingly, the PIAQ-based organic anode material displays excellent reversible specific capacity (1231 mAh/g at a current density of 200 mA/g), outstanding long-term cycle stability (its specific capacity was 486 mAh/g after 1000 cycles at the high current density of 1 A/g), and perfect rate performance. Both the excellent charge–discharge performance and perfect structural stability of PIAQ material make it one of the most prospective organic anode materials for next-generation high-performance LIBs.

A summary of the electrochemical properties of OCNs which were reported as anodes for LIBs is presented in Table 4.

## 7. Conclusions, Challenges, and Outlooks on Further Developments of Organic Anode Materials for LIBs

### 7.1. Challenges and Strategies for Enhancing the Electrochemical Performance of Organic Anode Materials for LIBs

Although organic electrode materials possess so many advantages [36,121], there are also several challenges impeding their commercialization, such as their intrinsic low electronic conductivity at room temperature that hinders the reaction kinetics of organic rechargeable batteries, uncontrollable side reactions, and high solubility in organic electrolytes, which leads to fast capacity fading [87,122,123]. Ingenious strategies, such as molecular engineering, nanosizing [78,124], modification, and combination with other materials, have been applied to alleviate the stated intrinsic drawbacks of organic electrode materials [60,80,125,126,127,128,129]. Moreover, a deeper insight into the reaction mechanisms of this type of material is also essential.

Some of the typical strategies to overcome the poor electronic conductivity problem of organic electrode materials are introducing conductive carbon additives (10–60%) [127], substituent group design, and adjusting the length of the carbon chain in an organic molecule.

As for the problem of dissolving organic compounds in common organic electrolytes, it can be overcome by using a solid polymer electrolyte. Besides, electrolyte optimization, regulating the structure of organic electrode materials by molecular engineering, and polymerization emerge as the main effective methods for inhibiting the dissolution of organic small molecules and enhancing the electrochemical performance of organic anode materials [130].

The nanosizing of organic materials in different shapes has been one of the most popular techniques for improving the electrochemical performance of organic electrode materials. For instance, Wang and co-workers produced Li_4_C_8_H_2_O_6_ in nanosheets and reported that its electrochemical properties excel those in bulk particles or in nanoparticles [124]. A nanosheet Li_4_C_8_H_2_O_6_ electrode displayed superior electrochemical performance with a first discharge capacity of 358 mAh/g as an anode material. It should be noted that the nanosheet Li_4_C_8_H_2_O_6_ electrode noticeably outperforms its bulk counterpart, exhibiting a discharge capacity of 175 mAh/g as an anode active material even at a high rate of 5C, which is approximately two times higher than those of the Li_4_C_8_H_2_O_6_ bulk. This outstanding electrochemical behavior of the Li_4_C_8_H_2_O_6_ nanosheet was attributed both to the 2D particle morphology causing a rapid lithium diffusion rate and to the larger surface area making lithium ions more accessible to each organic molecule serving as the active material of negative electrode of rechargeable LIBs. Overall, the findings demonstrated that Li_4_C_8_H_2_O_6_ nanosheets having multifunctional groups and high-specific surface area are perspectives in the applications of all organic LIBs [124].

Using lithium salts of organic molecules is also one of the effective strategies for inhibiting the undesired dissolution of organic electrode materials in aprotic electrolytes widely employed in LIBs and enhancing the cycling stability of organic anode materials for LIBs [68,71,131,132].

### 7.2. Outlooks on Further Developments of Organic Anode Materials for LIBs

Considering the aforementioned challenges, the emphasis of upcoming research on the development of high-performance organic anode materials for LIBs should be placed on the following aspects:

- It is well-known that the theoretical capacity of an active cathode or anode material is ultimately dependent on the number of electrons transferred in each redox-active moiety and inversely proportional to the molecular weight of the organic molecule. Since organic anode materials exhibit redox activity based on redox-active centers, there is a large proportion of inactive mass which inevitably decreases the reversible capacity of the negative electrode. Therefore, enhancing the density of active centers in any redox-active organic molecule is required to boost the capacity of the negative electrode. Although the molecular design can enhance the theoretical capacity of the organic anode material, several bulk properties of the anode material, such as crystallinity or particle size, influence the practical capacities.
- The search for unexplored redox-active functional groups in organic molecules continues to stimulate fundamental investigations. The early success of advanced porous materials, such as MOFs and COFs, towards rapid and stable cycling of Li-ions, is likely to accelerate the development of new organic anode materials for LIBs. Particularly, the optimal stability of MOFs or COFs in organic solvents may enable a robust pathway to utilize the features of organic redox into stable electrode materials.
- Apart from great efforts which have been made in molecular engineering to enhance the electrochemical parameters of organic anode materials, techniques to fabricate scalable organic anode materials are another aspect of further investigations. Expanding the scope of Li-ion battery technology will require reliable solutions for future energy needs. The investigations on such organic redox-active molecules are in their infancy and will require a comprehensive assessment of various classes of organic materials and compounds to fully realize their potential.

Thus, there is plenty of room to develop organic anode materials for LIBs with enhanced performance, such as long cycle life, high-output voltage, and high specific capacity.

Hopefully, this work provides a brief, useful introduction that outlines the representative organic anode materials in LIBs and gives new insight for the further application of organic anode materials for LIBs.

## Figures and Tables

**Table 1:** Structural formulas of typical organic anode materials based on carbonyl compounds and their key electrochemical parameters.

Organic Anode Material	Current Density/Specific Capacity (C or mAg^−1^/mAhg^−1^)	Structural Formula of Organic Active Anode Material	Refs.
Lithium perylene-3,4,9,10-tetracarboxylate (Li-PTCA)	24 mAg^−1^/195 (with 61.5% capacity retention after 50 cycles) 240 mAg^−1^/200 (with 60% capacity retention after 50 cycles)	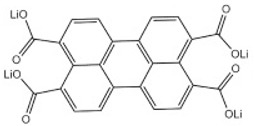	[29]
Lithium 2,6-bis(ethoxycarbonyl)-3,7-dioxo-3,7-dihydro-*s*-indacene-1,5-bis(olate)	0.05C/125	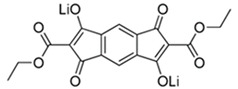	[68]
Lithium *trans-trans-*muconate	0.1C/170 (with 73.5% capacity retention after 80 cycles)	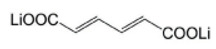	[71]
Lithium terephthalate	0.1C/300 (with 78% capacity retention after 50 cycles)	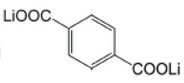	[71]
Alkaline earth metal terephthalates	200 mAg^−1^/130	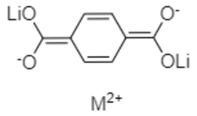 M = Ca, Sr, Ba	[73]
Lithium tannic acid (LiTA)	100 mAg^−1^/133.5 (with 75.3% capacity retention after 100 cycles)	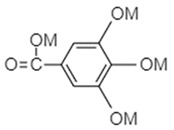 M = Li	[74]
Copper maleate hydrate (CMH)	200 mAg^−1^/404.6 (with 94.7% capacity retention after 250 cycles)	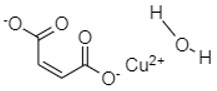	[75]
4-Nitrobenzoic acid lithium salt (NBALS)	0.5C (1C = 155 mAg^−1^) /153 (with 85.6% capacity retention after 100 cycles)	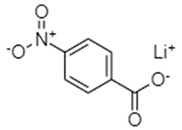	[76]
Dilithium 2,5-dibromoterephthalate (Li_2_-DBT)	0.1C/122, after 50 cycles	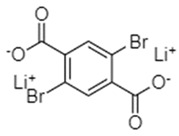	[77]
Dilithium 2,5-dimethoxyterephthalate (Li_2_-DMoT)	0.1C/95, after 50 cycles	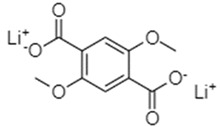	[77]
Dilithium 2,5-diaminoterephthalate (Li_2_-DAT)	0.1C/98, after 50 cycles	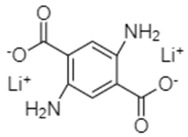	[77]

**Table 2:** Summary of the main electrochemical performance of various COF-based anode materials.

Organic Anode Material	Discharge Capacity or Capacity Retention (mAhg^−1^ or %)/Current Rate (mAg^−1^)/after Cycles	Discharge Capacities (mAh/g) with High Current Rate/Current Rate (mA/g)	Refs.
2D COF based-polyporphyrin (TThPP) film	381 mAhg^−1^ or 61.1%/1000/200	195/4000	[88]
N2-COF	600 mAhg^−1^ or 82%/1000/500	Unspecified	[89]
N3-COF	593 mAhg^−1^ or 81%/1000/500	Unspecified	[89]
Free-standing nitrogen-rich graphene-like holey conjugated polymers (NG-HCP nanosheets)	1015 mAhg^−1^/100/230	237/2500	[90]
Bulk covalent organic framework (COF)	125 mAhg^−1^/100/300	Unspecified	[91]
Two-dimensional covalent organic framework trapped by carbon nanotubes (COF@CNTs composite)	1536 mAhg^−1^/100/500	217/5C (1C = 1Ag^−1^)	[91]
E-CIN-1/CNT	1005 mAhg^−1^ or 79.2%/100/250	97/5000	[92]
E-SNW-1/CNT	920 mAhg^−1^ or 62.6%/100/250	212/5000	[92]
PA-TA	543 mAhg^−1^ or %/1000/400	207/5000	[93]

**Table 3:** Summary of the main electrochemical performance of Li-ion full cells assembled with MOF-based anode materials.

Organic Anode Material	MOF Template	Cathode Material	Current Density (C or mAg^−1^)/Specific Capacity (mAhg^−1^)/after Cycles/Capacity Retention (%)	Refs.
CoCOP	CoCOP	LFP	1C/69/300/83	[104]
Ni-IOHCs	Ni-IOHCs	LFP	0.1C/140/20/−	[105]
Fe-MIL-88B	Fe-MIL-88B	LFP	0.25C/86.8/100/73.7	[106]
Fe-MOF	Fe-MOF	LCO	500 mAg^−1^/120/1000/−	[107]
Cu-CAT	Cu-CAT	NCM811	200 mAg^−1^/371/200/−	[108]
[Zn_2_(py-TTF-py)_2_(BDC)_2_]·2DMF·H_2_O	(TTFs)-based Zn-MOF	NCM622	100 mAg^−1^/131.9/70/−	[109]
F-Co-MOF/rGO	F-Co-MOF	LFP	200 mAg^−1^/165.2/300/−	[110]
Si@Sn-MOF	Sn-based MOF	NCM622	20 mAg^−1^/117.7/150/87.8	[111]
Si@Cu_3_(HITP)_2_	Cu_3_(HITP)_2_	LCO	0.1C/1038/50/46	[112]
NiSb⊂CHSs	Ni-based MOF	LMO	200 mAg^−1^/228.2/100/−	[113]
Ni-Co-Sb/C	Ni-Co-MOF	LCO	100 mAg^−1^/354/100/53.2	[114]
m-Si HC-graphite	ZIF-8	LCO	0.2C/−/100/72	[115]
Co_3_O_4_@N-C	Co−MOF	LFP	100 mAg^−1^/266/100/95.3	[116]
Zn/Ni-Co-Oxide	Zn/Ni-ZIF-67	NCM532	1000 mAg^−1^/1060/80/70	[117]
Ni-MoO_2_@Co-CoO-NC	ZIF-67 polyhedron integrated with NiMoO_4_·xH_2_O nanowires	LFP	100 mAg^−1^/130/60/92	[118]

**Table 4:** Summary of the electrochemical properties of organic anode materials with nitrogen-containing groups employed as an anode for LIBs.

Organic Anode Material	Molecular Weight of Monomer	Anode Electrode Composition with the Mass Ratio (%)	Voltages of [Discharge]/[Charge] Platform or Average Voltages (V)	Initial Discharge (Lithiation) and Charge (Delithiation) Capacities (mAh/g)//at a Current Density (mA/g)	Discharge Capacity or Capacity Retention (mAhg^−1^ or %)/Current Rate (mAg^−1^)/After Cycles/Voltage Range vs. Li^+^/Li	Discharge Capacities (mAh/g) with High Current Rate/Current Rate (mA/g)	Refs.
2, 2′-Azobis(2-methylpropionitrile) (AIBN)	164	AIBN:super P carbon black:polyvinylidene fluoride = 80:10:10	[1.7, 1.2]/[-]	100/-//5	Unspecified	30/50	[54]
2,2′-Azobis(2-methylpropionamidine) dihydrochloride (AIBA)	198	AIBA:super P carbon black:polyvinylidene fluoride = 80:10:10	[1.7, 1.6, 0.6]/[-]	~1000/~160//5	110 mAhg-^1^/10/200/0.01–3 V	~50/50	[54]
Free-standing nitrogen-rich graphene-like holey conjugated polymers (NG-HCP nanosheets)	1405	NG-HCP nanosheets:carbon nanotubes:poly(vinylidene fluoride) = 70:20:10	[1.4]/[-]	2497/1319//20	1015 mAhg^−1^/100/230/0 ~3 V	237/2500	[90]
Bulk covalent organic framework (COF)	205	COF:acetylene black:polyvinylidene difluoride = 80:10:10	Unspecified	702/163//100	125 mAhg^−1^/100/300/0.005–3.0 V	Unspecified	[91]
Two-dimensional covalent organic framework trapped by carbon nanotubes (COF@CNTs composite)	205	COF@CNTs composite:acetylene black:polyvinylidene difluoride = 80:10:10	[1.5]/[0.75, 1.6]	928/383//100	1536 mAhg^−1^/100/500/0.005–3.0 V	217/5C (1C = 1Ag^−1^)	[91]
Polymeric Schiff base (PSB)	443	PSB:super P:polytetrafluoroethylene = 85:10:5	[0.15, 1.1, 2.3]/[0.3, 1.0, 3.2]	315/97//10	50.8%/10/More than 100/0.01–3.5 V	40.3/80	[119]
Poly(imine-anthraquinone) (PIAQ)	366	PIAQ:Ketjen black:Carboxymethylcellulose = 70:20:10	[1.0, 0.1]/[~1.3, ~0.3]	1231/1130//20	89.1%/200/More than 100/0.01–3.5 V	259/2000	[120]

## Data Availability

Not applicable.
